# Update on outpatient antibiotic prescribing during the COVID-19 pandemic: United States, 2020–2022

**DOI:** 10.1017/ash.2024.398

**Published:** 2024-10-31

**Authors:** Destani Bizune, Katryna Gouin, Lauren Powell, Adam L. Hersh, Lauri A. Hicks, Sarah Kabbani

**Affiliations:** 1 Medical Product Safety Branch, National Center for Emerging and Zoonotic Infectious Diseases, Centers for Disease Control and Prevention, Atlanta, Georgia; 2 Department of Pediatrics, Division of Infectious Diseases, University of Utah, Salt Lake City, UT, USA

## Abstract

We updated a descriptive analysis of national outpatient antibiotic prescribing during the COVID-19 pandemic. Prescribing volume was lower during 2020 and January–June in 2021 and 2022 compared to corresponding baseline months in 2019. Prescribing approached or exceeded baseline during July–December of 2021 and 2022 for all antibiotics, especially for azithromycin.

## Background

Most human antibiotic use in the United States (U.S.) is prescribed in traditional outpatient settings (including primary care offices and emergency departments), where viral respiratory infection visits account for a large proportion of unnecessary antibiotic prescribing.^1^ The coronavirus disease 2019 (COVID-19) pandemic impacted antibiotic prescribing patterns nationally; outpatient prescribing decreased significantly in 2020 compared to 2019.^2^ However, ongoing outpatient antibiotic use surveillance suggests continued use of azithromycin for patients with COVID-19, despite guidance recommending against the use of antibiotic therapy for non-critically ill patients.^3^ Few studies have examined national outpatient antibiotic prescribing patterns after 2020.^4^ The objective of this analysis is to provide an updated description of national outpatient antibiotic prescribing volume during the COVID-19 pandemic through 2022, highlighting the changes in azithromycin prescribing with changes in COVID-19 cases.^2^

## Methods

We used the IQVIA National Prescription Audit (NPA) Extended Insights dataset to identify all antibiotic prescriptions dispensed from US retail pharmacies during January 2019–December 2022. IQVIA collects data from approximately 48,900 US retail pharmacies that dispense 94% of all retail prescriptions and generates national projections of 100% of prescriptions. We reported monthly, quarterly, and annual oral antibiotic prescription volume. Prescribing rates per 1,000 persons were calculated using US Census yearly population estimates. Volumes and rates were stratified by patient age group (0–19, 20–64, and ≥65 yr) and antibiotic class. We calculated the percent change for each month and quarter from 2020–2022 compared to the baseline, defined as the corresponding month or quarter in 2019. Monthly prescription volume of azithromycin and COVID-19 cases was also described for the study period. Data on the number of COVID-19 cases were accessed through CDC’s COVID Data Tracker, which provides the monthly total of reported, laboratory-confirmed COVID-19 cases.^5^ This is a descriptive analysis that assessed overall directionality of change in prescribing over time. We conducted all analyses using Microsoft Excel and SAS 9.4 (SAS Institute, Cary, NC). This activity was reviewed by CDC and was conducted consistent with applicable federal law and CDC policy.

## Results

**Figure 1.**
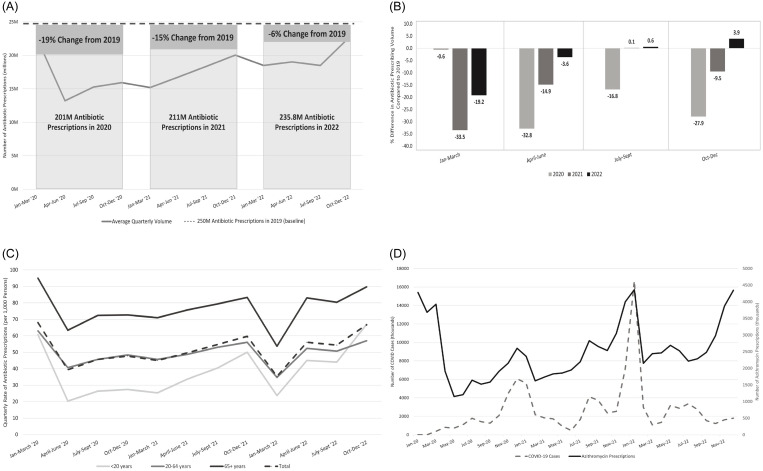
Antibiotic prescriptions dispensed from outpatient retail pharmacies in the United States in the National Prescription Audit Dataset, 2020–2022 (A) Antibiotic prescribing average quarterly volume and yearly percent change compared to 2019 baseline. (B) Percent difference in quarterly antibiotic prescribing volume compared to the corresponding 2019 baseline. (C) Quarterly rate of antibiotic prescriptions by age group, per 1,000 persons. (D) Volume of azithromycin prescriptions and reported COVID-19 cases in the United States, 2020–2022.

Compared to 2019, the total volume of antibiotic prescriptions decreased by 19.4% in 2020 (N = 201.3M), 15.4% in 2021 (N = 211.1M), and 5.5% in 2022 (N = 235.8M) (Figure 1A, Supplemental Table S1). While antibiotic prescribing remained lower than baseline from January 2020 to June 2021, prescription volume approached 2019 baseline levels from July to September 2021 (N =55M prescriptions, +0.1% change from baseline) (Figure 1B). From July to September 2022, antibiotic prescribing once again approached the 2019 baseline (N = 55M prescriptions, +0.6%) and exceeded baseline levels by 3.6% from October to December 2022 (N = 60M prescriptions) (Figure 1B).

Older adults (≥65 yr) had the highest quarterly prescribing rates throughout the study period, followed by adults (20–64 yr) and children (0–19 yr) (Figure 1C). The largest decrease in prescribing was observed in children, where in May 2020, children received 66% fewer antibiotic prescriptions than baseline, more than twice the decrease observed among adults and older adults (-32.9% and -31.9%, respectively) (Supplemental Table S1). Children had the largest increase in the quarterly prescribing rate from January-March 2021 to October-December 2021 (+100%) as compared to adults (+22%) and older adults (+17%) (Figure 1C). This continued from July to September 2022 through October to December 2022, where the quarterly prescribing rate in children exceeded the adult prescribing rate by 17% (57 per 1,000 in adults ≥20 yr vs. 67 per 1,000 in children) (Figure 1C).

Azithromycin prescriptions, which comprised 96%–97% of macrolide prescriptions, exceeded 2019 baseline levels by 11.0% in July 2021, 34.5% in August 2021, and 12.0% in July 2022 (Supplemental Table S3). From 2020 to 2022, trends in the volume of azithromycin prescriptions and COVID-19 cases were similar throughout most of the study period but particularly in 2021 (Figure 1D, Supplemental Table S3).

## Discussion

Using 2019 as a baseline, we found that the number of outpatient antibiotic prescriptions in the US declined during the COVID-19 pandemic; however, prescribing volume returned to and even exceeded baseline levels from July to December of 2022. The early decreases in antibiotic volume were mainly driven by decreased prescribing for children. Children also experienced the steepest increases in prescribing rates as prescribing returned to baseline. These findings are similar to a study characterizing antibiotic prescribing in primary care settings in the United Kingdom that found declines in prescribing at the onset of the pandemic and a return to “more normal prescribing trends.”^6^

Acute respiratory infections (ARIs) are a common cause of healthcare visits and antibiotic prescribing in outpatient settings and likely contributed to the observed trends in prescribing volumes.^1^ Increases in azithromycin prescriptions mirrored trends in reported COVID-19 cases throughout the pandemic, particularly in 2021. Higher rates of azithromycin prescribing compared with baseline may represent inappropriate prescribing for COVID-19 and other respiratory infections. Studies have shown that approximately 17% of persons diagnosed with COVID-19 in outpatient settings received an antibiotic, most commonly azithromycin.^7^ We observed an increase in prescribing for most antibiotic classes in 2022, particularly for macrolides, beta-lactams, cephalosporins, and tetracyclines. In late 2022, the US experienced a “triple epidemic” of influenza, respiratory syncytial virus, and COVID-19. The circulating respiratory viruses and the concurrent shortage of amoxicillin in the United States may have led to increased use of second-line antibiotics.^8^ During this same time period, the number of reported COVID-19 cases decreased while the number of azithromycin prescriptions increased, which may be partially due to increased use of home-based testing and prescribing for other respiratory infections.^9^

While the rates of outpatient antibiotic prescriptions have been decreasing since 2011, potentially reflecting targeted stewardship efforts, additional opportunities to avoid unnecessary prescribing still exist.^10^ The increased circulation of viral respiratory infections could lead to an increase in inappropriate antibiotic prescribing.^8^ Our findings highlight the need for enhanced stewardship efforts and support in the outpatient setting during respiratory virus season. Sustained improvement will require increased access to stewardship expertise and the implementation of multifaceted stewardship interventions adapted to urgent care and telemedicine settings. Providing stewardship implementation tools and resources can help improve prescribing practices.^11^ Optimizing the use of viral point-of-care and other diagnostic testing, such as Group A streptococcal disease tests, can decrease inappropriate prescribing for viral ARIs.^11^ Integrated health systems are expanding across the United States and can play an important role in supporting stewardship implementation in affiliated outpatient settings.^12^ CDC has increased funding to state and local health departments to maintain a local stewardship expert and support stewardship implementation in clinical settings, particularly those with limited access to stewardship expertise.

This study has several limitations. NPA data do not include diagnosis information; therefore, we were unable to assess prescribing indication. We were also unable to analyze other factors that may have contributed to changes in the volume or rate of outpatient antibiotic prescriptions by age group and antibiotic class, such as healthcare utilization or disease incidence. The reported incidence of COVID-19 cases was likely affected by the limited availability of diagnostic testing during 2020 and the increased availability of at-home testing in 2021 and 2022.

Antibiotic prescribing increased in the fall and winter of 2021 and 2022 across all age groups and returned to just below pre-pandemic levels, particularly among children. Overall antibiotic prescribing in 2022 was 6% lower than in 2019, and enhanced stewardship efforts are needed to maintain this downward trend. Optimizing vaccination and the testing and treatment of ARIs are important for patient safety and can lead to fewer outpatient visits resulting in an antibiotic.^11^ Ongoing surveillance and multifaceted antibiotic stewardship interventions are needed to optimize antibiotic use in outpatient settings beyond the COVID-19 pandemic.

## Supporting information

Bizune et al. supplementary material Bizune et al. supplementary material

## Supplementary material

For supplementary material accompanying this paper visit http://doi.org/10.1017/ash.2024.398. click here to view supplementary material
