# Supplementary material for: Update on outpatient antibiotic prescribing during the COVID-19 pandemic: United States, 2020–2022
Source: Antimicrob Steward Healthc Epidemiol. 2024 Oct 31;4(1):e193. doi: 10.1017/ash.2024.398 (PMC11526179; doi:10.1017/ash.2024.398)

Supplemental Table S1: Number, rate, and percent change (% Δ) of outpatient antibiotic prescriptions (℞) dispensed from retail pharmacies in the United States by age group, National Prescription Audit Dataset, 2020 – 2022

Year	Month	Total			Age[a]								
					0-19 Years			20-64 Years			≥65 Years		
		℞, No., in millions	℞ per 1000 persons, Rate	% Δ[b]	℞, No., in millions	℞ per 1000 persons, Rate	% Δ[b]	℞, No., in millions	℞ per 1000 persons, Rate	% Δ[b]	℞, No., in millions	℞ per 1000 persons, Rate	% Δ[b]
**2020**	January	24.3M	73	0.9	5.4M	65	1.3	13.0M	67	-0.3	5.9M	105	3.5
	February	22.1M	66	2.2	5.4M	65	1.0	11.6M	60	1.6	5.1M	90	5.0
	March	21.6M	65	-4.9	4.3M	52	-21.1	12.2M	62	1.1	5.1M	90	-1.8
	April	13.1M	39	-37.6	1.6M	20	-66.0	8.1M	41	-27.4	3.4M	59	33.2
	May	12.5M	37	-40.0	1.5M	19	-66.2	7.5M	38	-32.9	3.4M	61	-31.9
	June	14.1M	42	-20.9	1.8M	22	-47.0	8.3M	43	-16.0	3.9M	70	-11.8
	July	15.5M	46	-13.1	2.1M	26	-35.3	9.2M	47	-8.0	4.2M	74	-8.2
	August	15.0M	45	-17.5	2.1M	26	-40.3	8.9M	45	-12.2	4.0M	71	-11.4
	September	15.2M	46	-19.9	2.3M	27	-44.5	8.9M	45	-14.5	4.1M	72	-10.0
	October	16.1M	48	-23.3	2.4M	29	-48.7	9.5M	49	-16.6	4.3M	70	-14.9
	November	15.3M	46	-28.2	2.2M	27	-58.5	9.1M	46	-19.0	4.0M	70	-16.2
	December	16.4M	49	-32.2	2.2M	26	-63.8	9.8M	50	-23.1	4.4M	78	-19.2
	**Total**	**201.3M**	**602**	**-19.4**	**33.2M**	**402**	**-40.2**	**116.1M**	**594**	**-14.0**	**51.7M**	**917**	**-15.4**
**2021**	January	15.7M	46	-35.1	2.0M	24	-62.7	9.4M	48	-28.4	4.3M	74	-24.6
	February	13.7M	41	-36.6	1.9M	23	-64.2	8.1M	41	-29.4	3.7M	64	-23.4
	March	16.2M	48	-28.7	2.4M	29	-56.1	9.4M	48	-22.2	4.4M	75	-15.3
	April	16.3M	48	-22.3	2.6M	31	-45.2	9.3M	48	-16.2	4.3M	75	-13.9
	May	16.4M	49	-20.8	2.8M	34	-37.3	9.3M	47	-16.4	4.3M	73	-15.7
	June	17.6M	52	-1.5	3.0M	36	-12.6	10.0M	51	0.3	4.6M	79	3.0
	July	18.0M	53	0.5	3.1M	37	-6.0	10.2M	52	2.0	4.6M	79	2.2
	August	18.8M	56	3.1	3.4M	41	-5.4	10.7M	55	5.7	4.7M	81	4.1
	September	18.4M	55	-3.2	3.6M	43	-11.8	10.2M	52	-1.5	4.6M	78	0.9
	October	18.7M	56	-10.9	3.8M	46	-16.9	10.3M	52	-9.9	4.6M	80	-7.5
	November	19.9M	59	-6.3	4.4M	53	-16.9	10.8M	55	-3.9	4.7M	81	-0.4
	December	21.4M	64	-11.3	4.2M	51	-28.6	12.0M	61	-6.1	5.2M	89	-4.5
	**Total**	**211.1M**	**626**	**-15.4**	**37.2M**	**450**	**-33.1**	**119.6M**	**610**	**-11.8**	**54.1M**	**928**	**-14.3**
**2022**	January	20.0M	59	-16.9	3.0M	37	-42.7	11.7M	59	-10.8	5.3M	88	-7.2
	February	16.1M	47	-25.7	2.8M	34	-47.6	8.9M	45	-22.3	4.4M	73	-9.7
	March	19.3M	57	-15.0	3.8M	45	-30.3	10.4M	53	-13.7	5.1M	84	-2.1
	April	18.6M	55	-10.9	3.7M	45	-21.7	10.0M	51	-9.9	4.9M	81	-2.8
	May	19.5M	57	-6.1	4.0M	48	-11.1	10.4M	53	-6.5	5.0M	84	-0.4
	June	19.0M	56	6.1	3.5M	42	0.8	10.4M	53	4.6	5.1M	84	13.3

	July	17.3M	51	-2.9	2.9M	35	-11.0	9.7M	49	-3.5	4.7M	78	4.3
	August	18.8M	55	3.2	3.5M	43	-0.4	10.2M	52	0.9	5.0M	83	11.0
	September	19.3M	57	1.5	4.4M	54	8.9	10.0M	51	-3.6	4.8M	80	6.7
	October	21.1M	62	0.4	5.3M	64	15.4	10.7M	54	-6.0	5.1M	84	1.2
	November	23.1M	68	13.3	6.0M	72	13.1	11.7M	59	4.2	5.4M	89	13.3
	December	23.7M	70	-2.0	5.4M	65	-9.4	12.5M	63	-2.3	5.8M	96	7.0
	**Total**	**235.8M**	**694**	**-5.5**	**48.4M**	**583**	**-13.3**	**126.6M**	**644**	**-6.8**	**60.6M**	**1006**	**-7.1**

[a]Age unspecified not pictured but included in total

[b]Percent change from baseline, defined as corresponding month in 2019

Supplemental Table S2. Number, rate, and percent change (% Δ) of outpatient antibiotic prescriptions (℞) dispensed from retail pharmacies in the United States by antibiotic class, National Prescription Audit Dataset, 2020-2022																			
**Year**	**Month**	**Penicillins**			**Cephalosporins**			**Macrolides**			**B-lactams increased activity**			**Tetracyclines**			**Other**		
		**℞, No., in millions**	**℞ per 1000 persons, Rate**	**% Δ[a]**	**℞, No., in million s**	**℞ per 1000 persons, Rate**	**% Δ[a]**	**℞, No., in millions**	**℞ per 1000 persons, Rate**	**% Δ[a]**	**℞, No., in millions**	**℞ per 1000 persons, Rate**	**% Δ[a]**	**℞, No., in millions**	**℞ per 1000 persons, Rate**	**% Δ[a]**	**℞, No., in millions**	**℞ per 1000 persons, Rate**	**% Δ[a]**
**2020**	January	5.8M	17	0.0	3.4M	10	0.0	4.5M	13	-7.1	3.1M	9	0.0	2.4M	7	0.0	5.2M	16	-6.3
	February	5.5M	16	0.0	3.2M	9	0.0	3.8M	11	-8.3	2.7M	8	0.0	2.2M	6	0.0	4.7M	14	0.0
	March	4.8M	14	-17.6	3.0M	9	-10.0	4.1M	12	0.0	2.6M	8	0.0	2.2M	7	16.7	4.9M	15	0.0
	April	2.3M	7	-53.3	1.8M	6	-33.3	2.0M	6	-40.0	1.4M	4	-42.9	1.6M	5	-16.7	4.0M	12	-26.7
	May	2.4M	7	-53.3	2.0M	6	-33.3	1.2M	4	-55.6	1.2M	4	-42.9	1.6M	5	-16.7	4.1M	12	-26.7
	June	3.0M	9	-25.0	2.3M	7	-12.5	1.3M	4	-42.9	1.3M	4	-33.3	1.7M	5	-16.7	4.6M	14	0.0
	July	3.2M	10	-16.7	2.5M	7	-22.2	1.7M	5	-16.7	1.4M	4	-20.0	1.8M	5	-16.7	4.9M	15	-12.5
	August	3.1M	9	-25.0	2.4M	7	-22.2	1.6M	5	-28.6	1.4M	4	-20.0	1.7M	5	-16.7	4.8M	14	-12.5
	September	3.2M	10	-23.1	2.4M	7	-22.2	1.7M	5	-37.5	1.4M	4	-33.3	1.7M	5	-16.7	4.7M	14	-6.7
	October	3.4M	10	-33.3	2.5M	7	-22.2	2.0M	6	-40.0	1.5M	5	-28.6	1.9M	6	0.0	4.8M	14	-12.5
	November	3.1M	9	-40.0	2.3M	7	-22.2	2.3M	7	-36.4	1.5M	4	-50.0	1.8M	5	-16.7	4.4M	13	0.0
	December	3.3M	10	-41.2	2.3M	7	-30.0	2.7M	8	-42.9	1.6M	5	-44.4	1.9M	6	-14.3	4.6M	14	-6.7
	**Total**	**43.1M**	**129**	**-26.9**	**30.1M**	**90**	**-19.1**	**28.9M**	**87**	**-27.4**	**21.0M**	**63**	**-26.3**	**22.5M**	**67**	**-9.4**	**55.7M**	**33**	**-10.6**
**2021**	January	3.2M	10	-41.2	2.2M	7	-30.0	2.5M	7	-50.0	1.5M	4	-55.6	1.9M	6	-14.3	4.4M	13	-12.5
	February	3.0M	9	-43.8	2.0M	6	-33.3	1.7M	5	-58.3	1.3M	4	-50.0	1.7M	5	-16.7	4.0M	12	-21.4
	March	3.7M	11	-35.3	2.5M	7	-30.0	1.8M	5	-58.3	1.6M	5	-37.5	2.0M	6	0.0	4.7M	14	-6.7
	April	3.7M	11	-26.7	2.5M	7	-22.2	1.9M	6	-40.0	1.6M	5	-28.6	2.0M	6	0.0	4.5M	13	-6.7
	May	3.7M	11	-26.7	2.6M	8	-11.1	2.0M	6	-33.3	1.7M	5	-28.6	2.0M	6	0.0	4.5M	13	-6.7
	June	3.9M	12	0.0	2.8M	8	0.0	2.1M	6	-14.3	1.9M	6	0.0	2.2M	6	0.0	4.8M	14	0.0
	July	3.8M	11	-8.3	2.9M	9	0.0	2.3M	7	16.7	1.9M	6	20.0	2.1M	6	0.0	4.9M	15	-12.5
	August	4.0M	12	0.0	2.9M	9	0.0	2.9M	9	28.6	1.9M	6	20.0	2.2M	6	0.0	4.9M	15	-12.5
	September	4.0M	12	-7.7	2.8M	8	-11.1	2.8M	8	0.0	1.9M	6	0.0	2.1M	6	0.0	4.8M	14	-6.7
	October	4.2M	12	-20.0	2.9M	8	-11.1	2.6M	8	-20.0	2.1M	6	-14.3	2.1M	6	0.0	4.8M	14	-12.5
	November	4.6M	14	-6.7	2.9M	9	0.0	3.2M	9	-18.2	2.4M	7	-12.5	2.2M	7	16.7	4.6M	14	0.0
	December	4.6M	14	-17.6	3.0M	9	-10.0	4.1M	12	-14.3	2.6M	8	-11.1	2.4M	7	0.0	4.7M	14	-6.7
	**Total**	**46.4M**	**138**	**-22.0**	**32.0M**	**95**	**-14.7**	**29.9M**	**89**	**-25.6**	**22.5M**	**67**	**-21.4**	**24.7M**	**73**	**-1.1**	**55.6M**	**33**	**-11.3**
**2022**	January	2.3M	12	-29.4	2.6M	8	-20.0	4.5M	13	-7.1	3.9M	7	-22.2	4.4M	7	0.0	2.3M	13	-18.8

	February	1.9M	11	-31.3	2.4M	7	-22.2	2.2M	7	-41.7	3.6M	5	-37.5	4.1M	6	0.0	1.8M	12	-14.3
	March	2.2M	13	-23.5	2.9M	9	-10.0	2.6M	8	-33.3	4.6M	7	-12.5	4.7M	7	16.7	2.3M	14	-6.7
	April	2.2M	13	-13.3	2.8M	8	-11.1	2.6M	8	-20.0	4.4M	7	0.0	4.5M	6	0.0	2.3M	13	-20.0
	May	2.3M	13	-13.3	3.0M	9	0.0	2.8M	8	-11.1	4.5M	7	0.0	4.6M	7	16.7	2.3M	13	-6.7
	June	2.3M	12	0.0	3.0M	9	12.5	2.6M	8	14.3	4.2M	6	0.0	4.7M	7	16.7	2.2M	14	0.0
	July	2.1M	11	-8.3	2.8M	8	-11.1	2.3M	7	16.7	3.6M	6	20.0	4.6M	6	0.0	1.9M	14	-18.8
	August	2.2M	12	0.0	3.0M	9	0.0	2.4M	7	0.0	4.2M	6	20.0	5.0M	6	0.0	2.0M	15	-6.3
	September	2.1M	14	7.7	3.1M	9	0.0	2.6M	8	0.0	4.6M	6	0.0	4.7M	6	0.0	2.2M	14	-6.7
	October	2.3M	15	0.0	3.3M	10	11.1	3.1M	9	-10.0	5.2M	8	14.3	4.7M	7	16.7	2.6M	14	-12.5
	November	2.4M	15	0.0	3.6M	11	22.2	4.0M	12	9.1	5.3M	9	12.5	4.6M	7	16.7	3.2M	13	0.0
	December	2.6M	15	-11.8	3.4M	10	0.0	4.5M	13	-7.1	5.1M	10	11.1	4.6M	8	14.3	3.5M	14	-6.7
	**Total**	**53.1M**	**156**	**-11.3**	**35.9M**	**106**	**-5.0**	**36.1M**	**106**	**-10.9**	**28.6M**	**84**	**-0.8**	**26.9M**	**79**	**6.8**	**55.2M**	**32**	**-12.7**

[a]Percent change from baseline, defined as corresponding month in 2019

Supplemental Table S3. Number of COVID-19 cases and number, rate, and percent change (% Δ) of azithromycin prescriptions (℞), National Prescription Audit Dataset, 2020-2022

Year	Month	COVID-19 Cases	Azithromycin Prescriptions		
		Number, in thousands	Number, in thousands	℞ per 1000 persons, Rate	% Δ[a]
2020	January	4 K	4280 K	13	0.6
	February	4 K	3684 K	11	-1.1
	March	374 K	3924 K	12	5.9
	April	810 K	1915 K	6	-38.1
	May	723 K	1151 K	3	-61 .0
	June	1096 K	1207 K	4	-46.8
	July	1793 K	1641 K	5	-17.0

	August	1425 K	1524 K	5	-27.6
	September	1256 K	1584 K	5	-39.7
	October	2097 K	1911 K	6	-38.7
	November	4473 K	2154 K	6	-38.0
	December	6065 K	2601 K	8	-40.0
2021	January	5582 K	2360 K	7	-45.2
	February	2173 K	1622 K	5	-56.5
	March	1838 K	1732 K	5	-53.3
	April	1727 K	1833 K	5	-40.8
	May	927 K	1856 K	6	-37.0
	June	475 K	1947 K	6	-14.2
	July	1657 K	2195 K	7	11.0
	August	4117 K	2830 K	8	34.5
	September	3732 K	2657 K	8	1.1
	October	2373 K	2533 K	8	-18.8
	November	2537 K	3047 K	9	-12.2
	December	7159 K	3999 K	12	-7.8
2022	January	16648 K	4364 K	13	1.3
	February	2975 K	2150 K	6	-42.3
	March	1071 K	2441 K	7	-34.1
	April	1352 K	2464 K	7	-20.4
	May	3184 K	2691 K	8	-8.7
	June	2869 K	2529 K	7	11.5
	July	3361 K	2216 K	7	12.0
	August	2737 K	2287 K	7	8.7
	September	1532 K	2479 K	7	-5.7
	October	1215 K	2978 K	9	-4.5
	November	1623 K	3873 K	11	11.5
	December	1795 K	4346 K	13	0.2

[a]Baseline is corresponding month in 2019

Supplemental Figure 1. Annual volume of antibiotic prescriptions dispensed from retail pharmacies in the United States stratified by antibiotic class, National Prescription Audit Dataset, 2020-2022

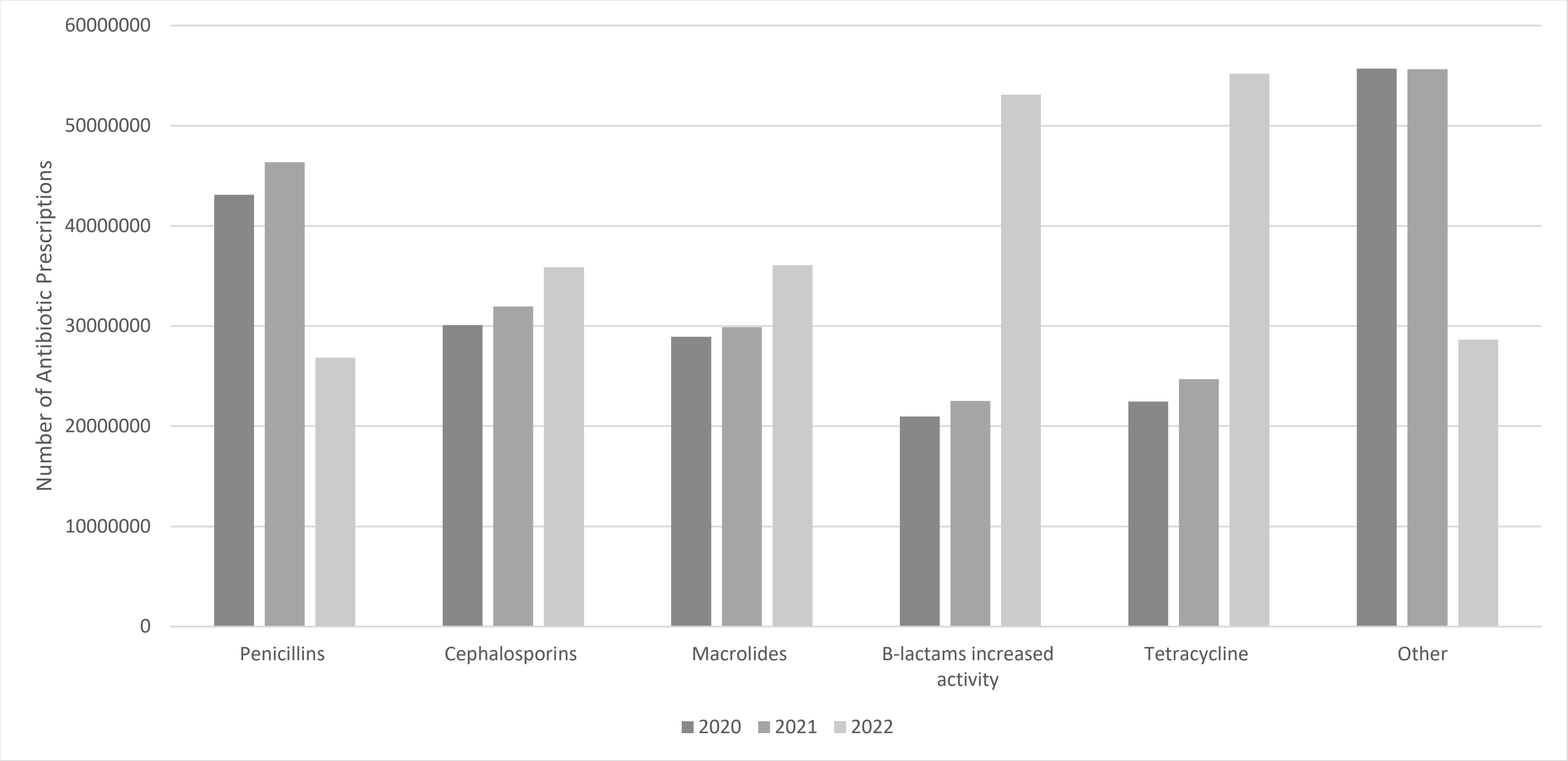

Supplement: Bizune et al. supplementary material [file S2732494X2400398Xsup001.pdf]
